# Heterogeneity of Adhesive Joint Properties

**DOI:** 10.3390/ma16237303

**Published:** 2023-11-24

**Authors:** Kamil Anasiewicz, Józef Kuczmaszewski

**Affiliations:** Faculty of Mechanical Engineering, Lublin University of Technology, 20-618 Lublin, Poland; j.kuczmaszewski@pollub.pl

**Keywords:** adhesive joint, nanoindentation, apparent Young’s modulus, adhesive joint material properties

## Abstract

This paper presents the results of a study of adhesive joints, focused on the heterogeneity of the properties of the adhesive material in the adhesive joint. The main objective of the study was to determine potential differences in the material properties of adhesive joints made with selected structural adhesives. Due to the impact of the joined material on the adhesive during the curing of the joint as well as the impact of phenomena occurring during the curing of the adhesive, the properties of the adhesive joint may vary along the thickness of the joint. Determining the differences in material properties over the thickness of the adhesive bond is important for more accurate prediction of adhesive bond strength in FEM simulations. In order to observe changes in the material properties of bonds, nanoindentation tests have been carried out on eight adhesive joint bonds made with common structural adhesives used to join sheets of aluminium alloy or corrosion-resistant steel. Basing on the achieved test results, load/unload curves were developed for imprints at characteristic spots of the joints. Distinct differences in the achieved average force value were observed for imprints located in the wall-adjacent zone and in the centre of the adhesive joint; this can be interpreted as areas of the joint with different material structures of higher or lower density of imperfections or porosities. Differences in the load/unload curves for ‘rigid’ and ‘flexible’ adhesives were analysed. The summary includes a conclusion that an adhesive joint is characterised by heterogeneous properties along its thickness.

## 1. Introduction

Adhesive bonding currently constitutes one of the most important and common methods of joining construction materials. It is one of the very few methods that allows different materials to be joined inseparably. Even though the first use of adhesive technology in joining materials dates back to 4000 BCE, the technology is constantly developing [1,2]. Said development of adhesive technology includes dynamic developments concerning adhesives, mixing and application methods, curing methods, and surface preparation for bonding. Despite the long-term evolution of bonding processes, certain phenomena accompanying bonding and their consequences still need to be clarified.

In previous research works by various authors, the topic of determining the adhesive properties of an adhesive joint has been addressed. The findings presented in literature studies show significant differences in Young’s modulus values of the adhesive in a joint, depending on the distance from the edge of the joint (connector-adhesive bond zone). There is a noticeable trend of decreasing Young’s modulus values with increasing distance from the surface of the connectors [3,4,5]. In other studies, authors have indicated the differences in Young’s modulus values of an adhesive joint and the sole adhesive material, made in a dumbbell shape, when subjected to axial tensile testing [6].

It is not uncommon for literature studies to address the issue of adhesive joint material properties [7,8] that are subject to modification. Often, the properties of an adhesive joint can be interpreted indirectly through the results obtained in the course of tests concerning the strength of the adhesive joints or material in paddle form [9]. However, more often than not, in their studies, authors usually interpret the adhesive joint material as homogeneous [10,11,12]. Few studies can be found in the literature that address the subject of differences in adhesive joint properties. In [13], the authors studied changes in Young’s modulus and hardness, using nanoindentation mapping, on wood adhesive joints. A slight decrease in Young’s modulus values and a significant reduction in hardness were observed as the bond moved away from the edge of the joint along the joint bond thickness. These changes were attributed to the penetration of the adhesive into the porosity of the wood connector. A similar scope of research was carried out in subsequent studies [14], and the authors additionally included numerical testing in their considerations and studied Young’s modulus and hardness depending on the imprint depth in the course of a nanoindentation study. Literature research papers often note differences in the material properties of the tested adhesives [15,16,17,18]. Other test results, also using nanoindentation, show significant differences in the values of Young’s modulus of samples in the form of adhesive joints and the same material made in the form of a dumbbell subjected to an axial tensile test. The Young’s modulus of an adhesive in a joint is higher by 85% in comparison to the base material [5].

In many research papers, the authors point out the correlation between adhesive layer thickness and adhesive connection strength, which may be considered a heterogeneity of adhesive joint material. Changes in the adhesive joint strength should be associated with an increased quantity of imperfections in the adhesive material occurring in the larger volume (thickness) of the adhesive joint. The inclusion of initial cracking in the design of the adhesive joint numerical model makes it possible to improve the accuracy of the results of numerical calculations compared to experimental results [19,20].

In view of the information presented in the above publications, it is important to consider if the bonding material may affect the mechanical properties of an adhesive in a joint.

The main objective of the presented research was to determine whether an adhesive joint could be considered a homogeneous material throughout its thickness or if differences in the properties of the adhesive joint material could be observed. The authors assumed the value of the force required to reach a fixed imprint depth in the nanoindentation test as an indicator of the heterogeneity of the joint material. The purpose was to determine whether there are significant differences in the load/unload characteristics obtained in the nanoindentation test between ‘rigid’ and ‘flexible’ construction adhesives. An equally important aspect of the conducted research is the comparison of load/unload characteristics concerning the characteristic measurement points of an adhesive joint. The characteristic points of an adhesive joint are defined by the authors as the points along the thickness of the adhesive joint where the greatest differences in material properties are expected. These points are symbolically marked as IF—joint wall-adjacent zone, i.e., the point closest to the edge of the joint, and C—joint core, i.e., the point in the middle of the joint.

## 2. Materials and Methods

In order to determine the load/unload characteristics for specific measurement points of adhesive joints, a test stand including a CSM Instruments ultrananoindenter (CSM Instruments, Needham Heights, MA, USA) was used. The precision measurement system of the CSM Instruments nanoindenter allows the indenter to be positioned with high accuracy. Results from the instrument measurement were developed using the Oliver–Pharr method, thanks to which it is possible to calculate Young’s modulus and the hardness of the material. The principle used in the Oliver–Pharr method consists of determining the stress–strain curves when performing a nanoindentation test and to apply the Hertzian mathematical model. This model describes the deformation of the material based on the geometrical parameters of the indenter and the elastic properties of the material [21].

For nanoindentation tests, eight samples were prepared: two for each of the Epidian 5 and Epidian 57 adhesive compositions [22], cured with PAC or Z1 hardeners, in the mass proportions recommended by the adhesive manufacturer, as presented in Table 1. The adhesives prepared in this way were used to join two construction materials: the first being the steel–steel configuration (1.4301 corrosion-resistant steel) and the second being the aluminium alloy–aluminium alloy configuration (EN AW 2024-T3).

The samples were made as a butt joint with initial dimensions of 40 × 60 × 6 mm. The sheets were initially cut using a hydroabrasive jet. The sheet metal surface was cleaned with ProfiSauber cleaner and Loctite 7061 degreaser. For the two materials to be joined, the surface preparation technology was different. The surface of the stainless steel sheet was sanded manually with 320-grit sandpaper, using circular motions in such a way as to produce a random directionality of irregularities on the surface of the prepared sheet. In the preparation of the aluminium alloy sheet surfaces, non-woven P320 abrasive sheets were used, and the surface was sanded by hand in such a way as to achieve a random distribution of irregularities on the metal sheet surface. Non-woven abrasive sheets were used for surface preparation due to the reduced clogging effect of non-woven material during operation compared to sandpaper, which results in more effective surface preparation. The surface of the sheets was then cleaned with a cleaner and twice with a Loctite 7061 degreaser. The excess of used materials was removed, and the sample was left to dry.

**Figure 1:**
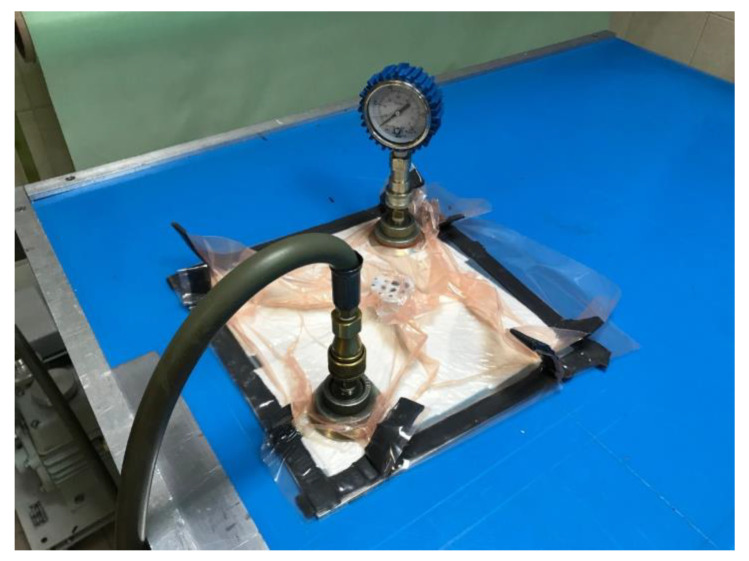
Samples for nanoindentation tests during the vacuum bag curing process.

The selected adhesive composition was applied to the cleaned surface of the sample with a spatula, spreading the adhesive evenly on both bonded surfaces. In the next step, the sheets were fixed relative to each other, the joint was secured against displacement, and it was placed in a specially prepared vacuum bag using a vacuum of 0.1 MPa. Due to the different densities of the used adhesive compositions, varying joint thicknesses were achieved. An attempt was made to obtain the lowest possible joint thicknesses in order to observe the strongest possible material ‘strengthening’ in the adhesive joint area. Samples were cured for a period of 24 h, under constant environmental conditions: pressure of 0.1 MPa, temperature 18–20 °C, and relative humidity 38–40%. Then, after removal from the vacuum bag, they were seasoned for a minimum of 168 h. Figure 1 presents a photo of preparing samples in a vacuum bag for nanoindentation tests.

**Figure 2:**
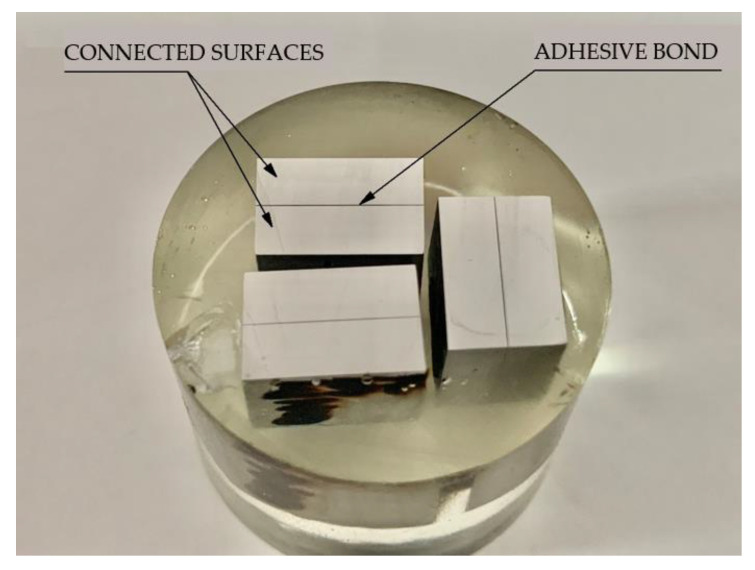
Adhesive joint sections prepared for nanoindentation tests.

After a seasoning period, 10 × 15 mm samples were cut from the centre of the sheet using the hydroabrasive jet cutting process for nanoindentation tests. Hydroabrasive jet cutting allows adhesive joint samples to be made, reducing the risk of bond destruction to a minimum, thanks to the lack of thermal effects occurring in the cutting zone. Samples prepared in such a way, with the surface exposed for testing, were placed in moulds and poured with Scandiplex resin. Samples with adhesive joint sections were prepared by pre-grinding on grinding discs while intensive cooling was applied. The surface preparation process continued with the use of abrasive papers, starting with coarse-grained paper (180, 240), and very fine machining was carried out using fine-grained papers (1000, 1200, 2500). During grinding, conditions were maintained to minimise the thermal impact on the adhesive joint by using an emulsion coolant. After grinding, the samples were subjected to mechanical polishing on horizontally arranged felt-lined rotating discs and covered with an Al_2_O_3_ water suspension. The polishing was carried out until it achieved a mirror-like, scratch-free surface. The finished sample was washed in water and ethanol and then dried with a stream of compressed air. Figure 2 presents a photograph of samples with adhesive joint sections prepared for nanoindentation tests.

**Figure 3:**
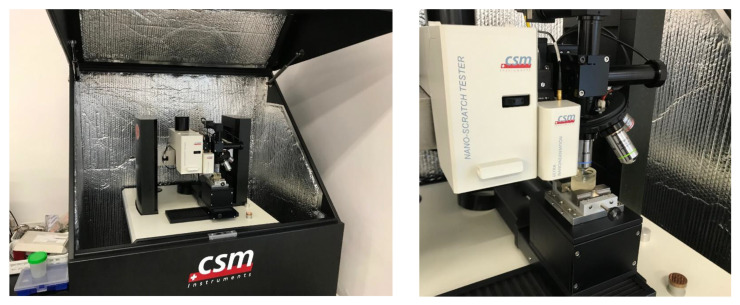
Research station with a CSM Instruments ultrananoindentometer.

This study, which aimed to observe the heterogeneity of material properties at the thickness of the adhesive joint depending on the distance from the phase boundaries, was carried out using a CSM Instruments ultrananoindenter. Figure 3 presents the test stand with a mounted sample.

The test was carried out on the thickness of the adhesive joint, starting from the metal–adhesive phase boundary to the centre of the joint, using a reference head to keep the indenter recesses constant in relation to the material surface. The indentations were made at two points, starting at a distance of 3 μm from the edge of the joint and at the center of the joint. In order to ensure averaging of the results, 10 repetitions were performed for each measurement point at various locations of the adhesive joint. Nanoindentation was performed using a diamond indenter with Berkovich geometry to a fixed depth of 800 nm.

## 3. Results

**Figure 4:**
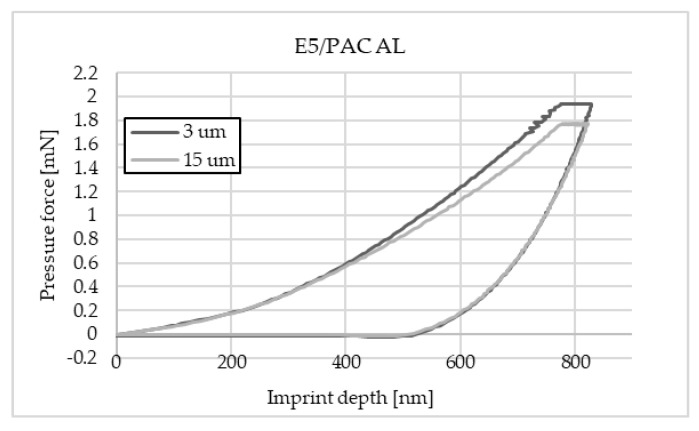
A graph of the course of measurement with a nanoindenter at a distance of 3 μm and 15 μm from the edge of the adhesive joint (phase boundary) for 0.030 mm thick E5/PAC epoxy adhesive bonding EN-AW 2024-T3 aluminium alloy sheets.

**Figure 5:**
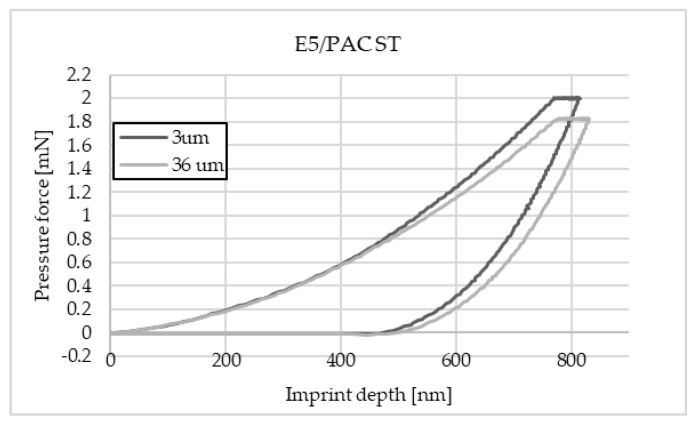
A graph of the course of measurement with a nanoindenter at a distance of 3 μm and 36 μm from the edge of the adhesive joint (phase boundary) for 0.072 mm thick E57/PAC epoxy adhesive bonding sheets of 1.4301 steel.

**Figure 6:**
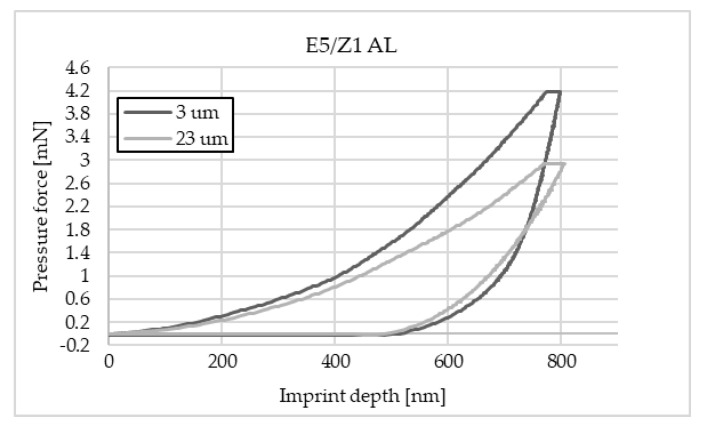
A graph of the course of measurement with a nanoindenter at a distance of 3 μm and 23 μm from the edge of the adhesive joint (phase boundary) for 0.046 mm thick E5/Z1 epoxy adhesive bonding EN-AW 2024-T3 aluminium alloy sheets.

**Figure 7:**
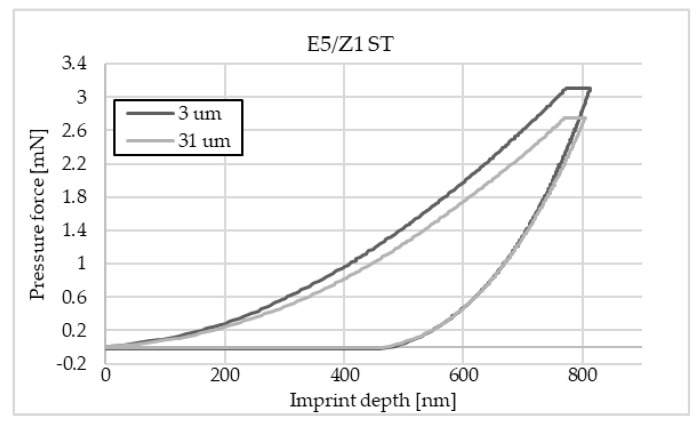
A graph of the course of measurement with a nanoindenter at a distance of 3 μm and 31 μm from the edge of the adhesive joint (phase boundary) for 0.062 mm thick E5/Z1 epoxy adhesive bonding sheets of 1.4301 steel.

**Figure 8:**
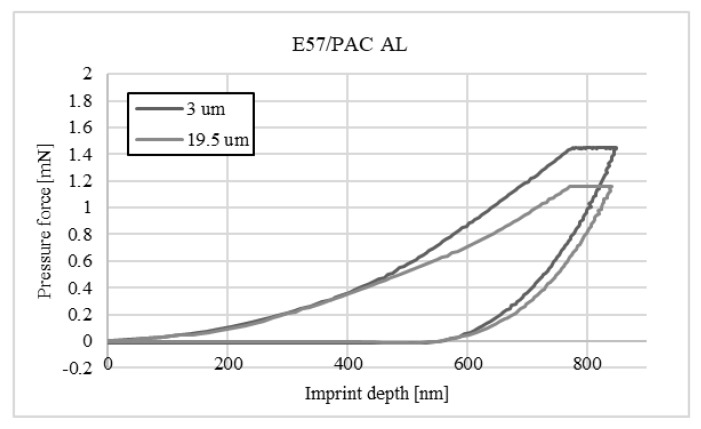
A graph of the course of measurement with a nanoindenter at 3 μm and 19.5 μm from the edge of the adhesive joint (phase boundary) for 0.039 mm thick E57/PAC epoxy adhesive bonding aluminium alloy sheets.

**Figure 9:**
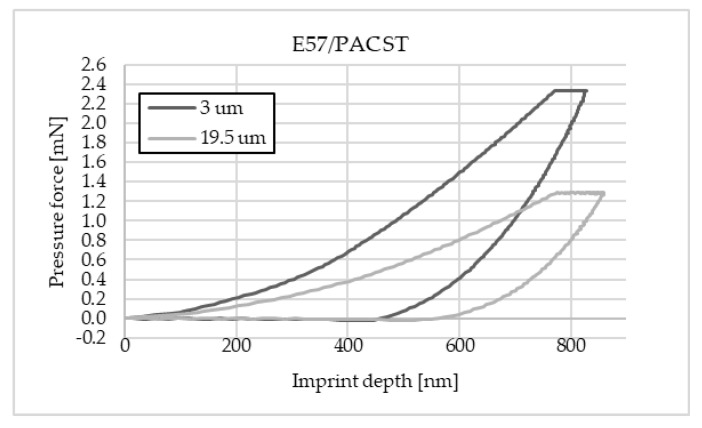
A graph of the course of measurement with a nanoindenter at a distance of 3 μm and 19.5 μm from the edge of the adhesive joint (phase boundary) for 0.039 mm thick E57/PAC epoxy adhesive bonding sheets of 1.4301 steel.

**Figure 10:**
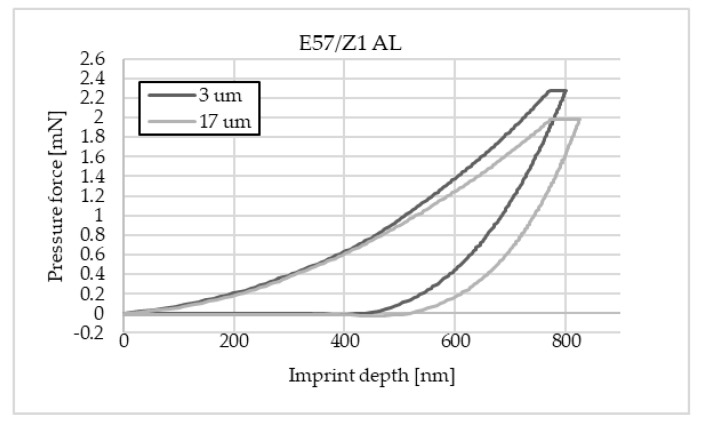
A graph of the course of measurement with a nanoindenter at a distance of 3 μm and 17 μm from the edge of the adhesive joint (phase boundary) for 0.034 mm thick Epidian 57/Z1 epoxy adhesive bonding aluminium alloy sheets.

**Figure 11:**
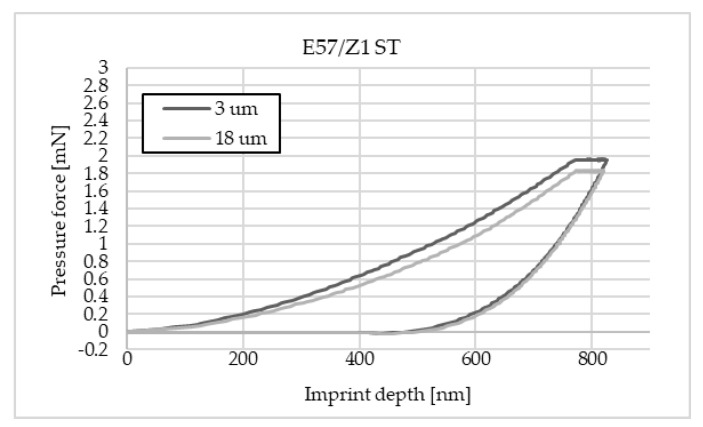
A graph of the course of measurement with a nanoindenter at a distance of 3 μm and 18 μm from the edge of the adhesive joint (phase boundary) for 0.036 mm thick Epidian 57/Z1 epoxy adhesive bonding sheets of 1.4301 steel.

The charts below (Figure 4, Figure 5, Figure 6, Figure 7, Figure 8, Figure 9, Figure 10 and Figure 11) present the course of the nanoindentation test for two characteristic measurement points: the point closest to the phase boundary—IF—and the point in the centre of the adhesive joint—C. We identified these points in turn with the wall-adjacent zone and the core of the adhesive joint. These are zones with potentially numerous joint material properties. The measurement point in the wall-adjacent zone was taken at a distance of 3 μm from the metal–adhesive border each time. The charts for the two joined materials are labelled AL for aluminium alloy and ST for corrosion-resistant steel. In the graph, starting from the left, it is possible to determine the loading curve, then the horizontal stabilisation curve located where the target imprint depth of 800 nm is reached, and the unloading curve, so that force hysteresis as a function of imprint depth can be observed. The presented curves constitute examples of representative curves obtained in the course of the nanoindentation test. This means that the curves selected for comparison were those that, for a given measurement point in the study, reached the value closest to the mean value from all the imprints taken after rejecting extreme values.

Comparing the curves for the Epidian 5/PAC flexible adhesive, it is possible to notice a higher value of force when making an imprint in the wall-adjacent zone. This results from the greater resistance when inserting the indenter into the material in this zone. The course of the curves at representative points of the joint is standard for this type of test. This means that the material at the point of measurement is homogeneous. No significant differences in the curves were observed between the joints that bind two sheets of aluminium alloy and corrosion-resistant steel.

Table 2 presents the average values of the maximum force obtained for nanoindentation. The values are provided for all tested adhesives for values in the wall-adjacent zone IF and the core (centre) of the joint C. The mass proportions used in the adhesive composition used for the joints are given next to the name of the hardener.

**Figure 12:**
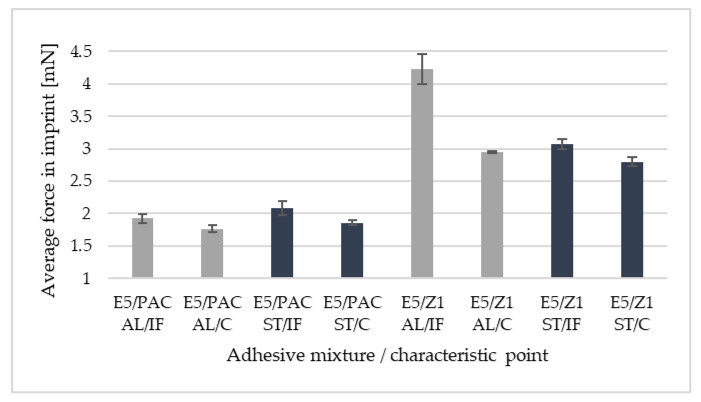
Comparison of average force values in imprint (800 nm) for Epidian 5 resin adhesives for two hardeners and two characteristic points: IF—wall-adjacent zone; C—joint core.

**Figure 13:**
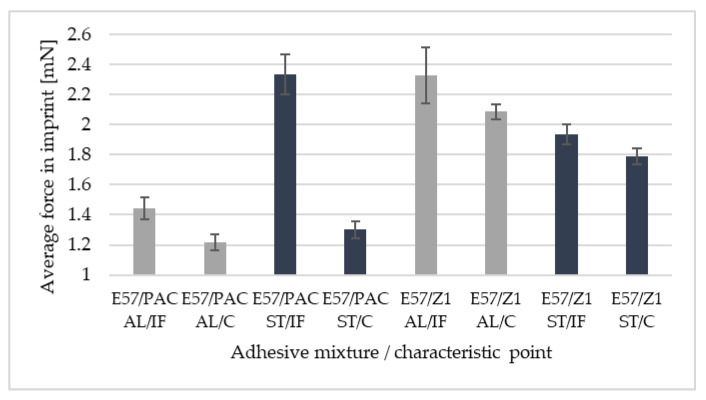
Comparison of the average force values in imprint (800 nm) for Epidian 57 resin adhesives for two hardeners and two characteristic points: IF—wall-adjacent zone; C—joint core.

**Figure 14:**
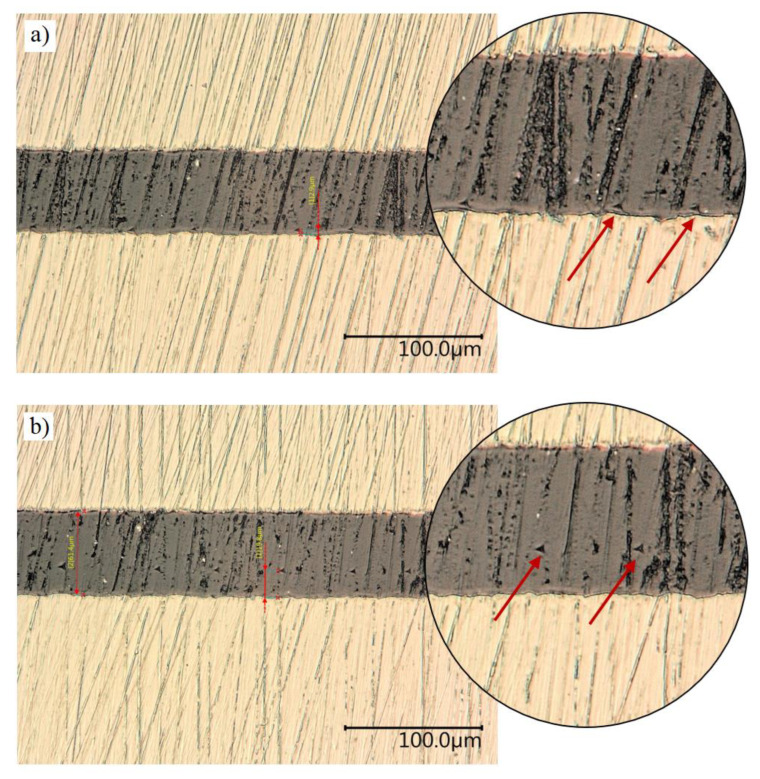
Imprints, indicated with arrow, in the adhesive joint material after nanoindentation testing: (**a**) imprints at 3 μm; (**b**) imprints at 19.8 μm.

Figure 12 and Figure 13 provide a comparison of the average force values at the maximum imprint depth, i.e., 800 nm. It is possible to notice clear differences in the force values for the characteristic points, i.e., the wall-adjacent zone IF and joint core C. The symbol AL denotes an aluminium alloy sample; ST denotes a 1.4301 steel sample. Figure 14 presents microscopic images of the sections, with the adhesive joint exposed and the imprint visible. The imprints are made at defined distances from the edge of the joint so that they do not interact with each other during the test.

In the provided microscope images, it is possible to see the individual imprints made in the joint material. They have been marked with arrows. The precision of performing individual imprints in relation to joint thickness should be emphasised. The precise measurement system of the CSM Instruments nanoindenter allows the indenter to be positioned at a resolution of 0.1 nm.

## 4. Discussion

In samples bonded with adhesives conventionally described as ‘rigid’, differences in the slope of the loading curve can be observed as compared to ‘flexible’ adhesives. The slope of the curve, obtained for rigid adhesives, is closer to the ordinate axis, while at the same time, the target depth of 800 nm is reached at a higher contact force. It is possible to formulate a general conclusion that the shape of the graph, and more precisely, its loading section, takes a shape closer to a straight line for rigid adhesives in comparison with that of flexible adhesives. However, this is true for curves at measuring points in the centre of the joint. It should also be noted that, in each of the analysed joints, significant differences were observed in the value of the maximum average force in the imprint, comparing the values obtained for the measurement points closest to the interphase zone and those in the centre of the adhesive joint. These differences range from 8.1% to 79.2%. A correlation was also determined between the selected measurement point and the standard deviation. For measuring points located in the centre of the joint, smaller scatterings of the measured values were observed. This may indicate that the adhesive joint is more homogeneous in the core area of the joint than in the adjacent area. It is not possible to unequivocally state that the bonded materials presented in the study have a significant effect on the maximum force values obtained in the imprints. If one should compare the differences between the characteristic measurement points (IF—wall-adjacent zone and C—joint core) in the samples bonding 1.4301 steel, they are on average greater (27.3%) than in the samples bonding EN-AW 2024-T3 aluminium alloy (20.6%). It should also be emphasised that the average differences in imprint force values between IF and C points, are greater for flexible adhesives (with PAC hardener) than for rigid adhesives (with Z1 hardener), regardless of the bonded material.

The obtained test results allow us to conclude that there are differences in the properties of the adhesive joint, which can be described as heterogeneity of the properties of the adhesive material along the thickness of the joint. As a measure of the heterogeneity of the joint material, the value of the force required to reach a fixed imprint depth in the nanoindentation test was used. In the presented comparative graphs, covering the entire range of studied adhesives, these differences form a recurring trend.

## 5. Conclusions

The experimental research presented here focused on determining the differences in material properties at the thickness of an adhesive joint connecting different structural materials. In particular, the differences in force values obtained in the imprints at the characteristic points of the joint were compared.

The results discussed support the following conclusions:

- The material in an adhesive joint exhibits different material properties which vary along its thickness.
- The material in the adhesive joint in the adjacent zone (IF) exhibits greater resistance during indentation than the material in the centre of the adhesive joint (C), which can be interpreted as local changes in material properties.
- As the thickness of the adhesive joint increases, greater differences are observed between the wall-adjacent zone and the core of the adhesive joint.
- The differences in the average force values in the nanoindentation imprints between the characteristic points can be interpreted as areas of the joint with different material structures of higher or lower density of imperfections, porosity, etc.
- Depending on the thickness of the adhesive joint, these differences may be important for the load-bearing capacity of the adhesive joint in an adhesive bond.
- The presented results should provide an important basis for improving the prediction of the strength of adhesive joints and, in particular, as part of the input data determining the variable material properties of the adhesive in the joint, e.g., stiffness required in the development of an improved numerical FEM model.

## Figures and Tables

**Table 1:** Mechanical properties and resin/curing agent mass ratios of used adhesive compositions.

Adhesive Composition	Resin/Curing Agent Mass Ratio	Young’s Modulus [MPa]	Tensile Strength [MPa]
Epidian 5/PAC	100:80	1285	50
Epidian 5/Z1	100:12	2029	70
Epidian 57/PAC	100:65	979	35
Epidian 57/Z1	100:10	1701	50

**Table 2:** Average values of maximum force in the analysed nanoindentation imprints.

		E5						E57					
		PAC (80 g/100 g)			Z1 (12 g/100 g)			PAC (65 g/100 g)			Z1 (10 g/100 g)		
Bonded Material	Item	Average Force [mN]	Standard Deviation [mN]	Joint Thickness [mm]	Average Force [mN]	Standard Deviation [mN]	Joint Thickness [mm]	Average Force [mN]	Standard Deviation [mN]	Joint Thickness [mm]	Average Force [mN]	Standard Deviation [mN]	Joint Thickness [mm]
AL	IF	1.923	0.1457	0.03	4.227	0.4528	0.046	1.443	0.1466	0.039	2.329	0.3763	0.034
	C	1.766	0.1034		2.948	0.0293		1.216	0.1075		2.086	0.1009	
ST	IF	2.085	0.2111	0.072	3.072	0.1465	0.061	2.332	0.2649	0.039	1.935	0.1381	0.036
	C	1.8583	0.0822		2.798	0.1434		1.301	0.1156		1.790	0.1092	

## Data Availability

The data presented in this study are available on request from the corresponding author.
